# Plant growth regulation by seed coating with films of alginate and auxin-intercalated layered double hydroxides

**DOI:** 10.3762/bjnano.11.93

**Published:** 2020-07-24

**Authors:** Vander A de Castro, Valber G O Duarte, Danúbia A C Nobre, Geraldo H Silva, Vera R L Constantino, Frederico G Pinto, Willian R Macedo, Jairo Tronto

**Affiliations:** 1 Laboratório de Compostos Lamelares (LCL), Universidade Federal de Viçosa, Campus de Rio Paranaíba, Rodovia MG-230, Km 7, CEP 38810-000, Rio Paranaíba-MG, Brazil; 2 Laboratório de Fisiologia e Metabolismo da Produção Vegetal (LAFIMEPRO), Universidade Federal de Viçosa, Campus de Rio Paranaíba, Rodovia MG-230, Km 7, CEP 38810-000, Rio Paranaíba-MG, Brazil; 3 Departamento de Química Fundamental, Instituto de Química, Universidade de São Paulo, Avenida Professor Lineu Prestes, 748, CEP 05508-000, São Paulo-SP, Brazil

**Keywords:** bioassays, intercalation compounds, layered double hydroxides (LDHs), layered materials, 1-naphthaleneacetic acid (NAA), plant-growth regulators

## Abstract

Auxins are a class of organic substances known as plant-growth regulators, which act on plant physiology, promoting its full development. However, due to the great instability of these substances among the diversity of crops and cultivation environments, it is necessary to seek more efficient modes of application, which lead to a homogeneous distribution and promote a sustained release according to the plants demand. Seed coating, using films containing a biodegradable polymer and auxins intercalated into layered compounds, emerges as a very promising approach to a new form of growth regulator application. Thus, the presented work had three aims: (i) the synthesis and characterization of an organic–inorganic hybrid material containing a layered double hydroxide (LDH) of zinc and aluminum and the synthetic auxin 1-naphthalenoacetic acid (ZnAl-NAA-LDH), (ii) the coating of bean seeds (*Phaseolus vulgaris L.*) with composite films produced from mixtures of alginate polymer and ZnAl-NAA-LDH, and (iii) the evaluation of the plant response by bioassays. The hybrid ZnAl-NAA-LDH was characterized by a set of analytical techniques, including powder X-ray diffraction, thermogravimetric analysis coupled to differential scanning calorimetry and mass spectrometry, specific surface area measurement, and scanning electron microscopy. Bioassays were performed with the seeds coated with the composite film to assess the germination rate and germination speed index of the seeds, as well as biometric analyses including measurements of root area, root fresh matter, and shoot length of the plants. The bioassay performed in soil pots showed that the alginate film containing ZnAl-NAA-LDH yields an enhancement regarding root area, fresh root matter and shoot length of plants. Thus, films produced from a mixture of alginate and the hybrid material containing the growth regulator intercalated into LDH can be a viable alternative to enhance plant development, which can be included in seed management.

## Introduction

Increasing food production in line with environmental preservation is a challenge to agricultural innovation. With regard to this, nanotechnology enables the development of new sustainable products to increase crop yields. Some studies have demonstrated the use of nanomaterials as host matrices for storage and slow release of active products as fertilizers and agrochemicals [1–6].

The intercalation of ions or molecules of agricultural interest into layered double hydroxides (LDHs) is an alternative to optimize the supply of these compounds to plants. Studies have demonstrated the application of LDHs as slow-release matrices for agrochemicals and fertilizers [7–11]. To increase the effectiveness of pest control, agrochemicals are often applied to plantations in higher doses than recommended. These high doses can cause environmental problems such as contamination of soil, water, plants, and animals. The intercalation of agrochemicals in LDHs can generate a sustained release of the organic compounds, reducing the number of applications and minimizing environmental risks. LDHs were intercalated with the herbicides (2,4-dichlorophenoxy)acetic acid, (4-chloro-2-methylphenoxy)acetic acid, and Picloram [9]. The slow release of the herbicides was evaluated in batch and column leaching tests. In addition, herbicidal activity was also tested in bioassays using cress (*Lepidium sativum*) as indicator plant. Compared to commercial herbicides, the application of LDHs reduced the maximum concentration of herbicides in the leachate causing a delay in the herbicide leaching by the soil. LDHs showed a herbicidal efficacy similar to that of commercial herbicides indicating the potential use of these materials as a support for the preparation of slow-release formulations of acidic herbicides.

A LDH intercalated with phosphate (LDH-phosphate) was synthesized and employed as slow-release fertilizer for the enhancement of phosphate fertilization efficiency, which is of particular relevance for tropical weathered soils [10]. A kinetic study of phosphorus release and a bioassay under controlled conditions using maize (*Zea mays*) as indicator plant were performed to test the suitability of the proposed material. The results obtained using LDH-phosphate were compared to those of commercial triple superphosphate (TSP) fertilizer in two different soils, namely, a sandy soil and a clayey soil. Under the experimental conditions of the bioassay, plant productivity, height, and the content of phosphorus in the dry matter increased as a result of applying the new LDH-phosphate fertilizer. Furthermore, the application of LDH-phosphate yielded an increased pH value of the soil, thus contributing to the decrease of the phosphorus adsorption capacity of the soil, making this nutrient more available to the plants. These results confirmed that LDH-phosphate has a great potential for the application as a new technology in phosphate fertilizers.

Boron is an important micronutrient for the growth of plants. It is generally present in soils as H_3_BO_3_. This acid exhibits weak retention in the soil and has a high rate of leaching. Castro et al. [11] synthetized alginate microspheres containing LDH intercalated with borate anions (LDH-B-ALG) and used them as a new slow-release boron fertilizer. In vitro release tests and leaching experiments showed that the boron release in the solution and the leaching in soil columns were much lower from LDH-B-ALG when compared to conventional boron sources. In the bioassays, the lower release and leaching from LDH-B-ALG improved the accumulation of boron in the root zone of the indicator plant, sunflower (*Helianthus annuus*), with consequent increase of fertilizer efficiency and uptake of boron by plants. The new fertilizer showed to be a suitable boron source for the growth of plants, especially in sandy soils.

The abovementioned works show that organic–inorganic hybrid materials provide a physical and chemical protection for the intercalated molecules. When these materials are applied, they can offer modified release of intercalated species and/or greater tolerance to biotic and abiotic factors, improving the physiological processes of plants.

Auxins are plant-growth regulators (PGRs), influencing physiological processes from embryogenesis to the formation of new organs [12]. The main auxins that favor plant growth are 1-naphthaleneacetic acid (NAA), (indol-3-yl)acetic acid (IAA) and 4-(indol3-yl)butyric acid (IBA) [13]. Today, the application of auxins in agriculture provides advantages such as longer time of action and stability of active agents, the minimization of the effects of biotic and abiotic stresses, and the control of diffusion, reaction rates, and other physicochemical parameters. However, because these substances are sensitive to light and/or temperature variations, their effectiveness for plant growth can be compromised by physical and chemical changes of the medium [13–15]. Searching for tools that improve the action of auxin in plants, we examined in this work the intercalation of NAA in inorganic layers of LDHs, with the subsequent production of a composite film with alginate [16–17] to provide physical and chemical protection for the intercalated auxin substances.

It is expected that the application of this material in agriculture promotes a modified release of the intercalated species and/or a greater auxin stability against the biotic and abiotic factors of the medium, since the synthesized nanocomposite ZnAl-NAA-LDH was added to the seed in a single step together with the polymeric coating with alginate. This is a different process from those reported in most studies in which the application of this method occurs in two distinct stages [18].

LDHs are two-dimensionally structured materials similar to brucite, Mg(OH)_2_, but having hydrated anions located between the layers due to the isomorphic substitution of bivalent cations (M^II^) by trivalent cations (M^III^). These materials can be described by the general formula [M^II^_1-_*_x_*M^III^*_x_*(OH)_2_]A*^n^*^−^*_x_*_/_*_n_*·*m*H_2_O, where A*^n^*^−^ is an anion with electric charge *n^−^*, and *m* denotes the number of water molecules [19–25].

Hussein et al. [15] intercalated NAA into ZnAl-LDH (Zn/Al molar ratio = 3.5) by co-precipitation and performed in vitro release studies. The anion release rate depended on the pH value of the medium. From the beginning of the experiment up to 8 h, the release of 23.2% and 22.0% of NAA in solutions of initially pH 1 and pH 7, respectively, followed a ﬁrst-order kinetic. In neutral pH solution, a release of 75.0% of NAA was verified after 168 h and the inorganic layered structure was kept integral in the first 7 days of the experiment.

Li et al. [26] synthesized two MgAl-LDHs (Mg/Al molar ratio = 2.0) intercalated with NAA and IBA, and evaluated the influence of temperature, pH value, and electrolyte solutions on the in vitro release. At a temperature of 25 °C, the release of NAA and IBA was quick in the first 100 min, followed by a gradual release profile. The increase in temperature led to a higher release of NAA and IBA, indicating that the release process was endothermic. The total released amounts of NAA and IBA at pH 4 and pH 12 were higher than the quantities released at pH 7. The study of electrolytes evidenced that the released quantities of NAA and IBA depend on the interfering anion present in the aqueous solution (CO_3_^2−^ ≈ SO_4_^2−^ > Cl^−^). According to the authors, this is related to the ion exchange capacity of the interfering anions.

To the best of our knowledge, this is the first study reporting the direct application of an organic–inorganic hybrid with NAA intercalated into a LDH in the evaluation of plant growth. This work describes (i) the synthesis and characterization of a zinc–aluminium LDH intercalated with NAA (ZnAl-NAA-LDH); (ii) the preparation a hybrid films for bean seed coating (*Phaseolus vulgaris L.*) containing a mixture of sodium alginate and ZnAl-NAA-LDH; (iii) bioassays with the coated seeds to evaluate germination rate and germination speed index (GSI), and biometric analyses with the values of root area, root fresh matter, and shoot length of the plants.

## Results and Discussion

### Characterization of ZnAl-NAA-LDH

**Figure 1:**
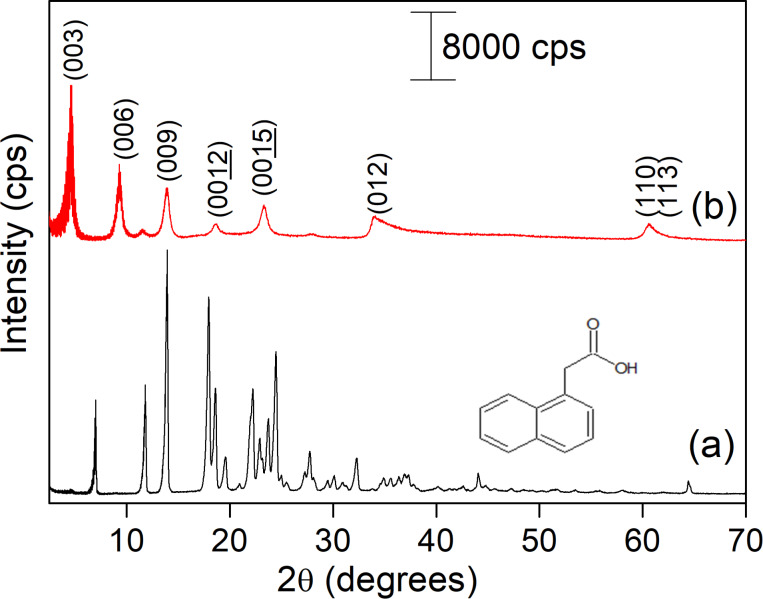
XRD patterns of (a) NAA and (b) ZnAl-NAA-LDH.

The powder X-ray diffraction (XRD) patterns of neat NAA and ZnAl-NAA-LDH are shown in Figure 1. The presence of the basal reflections (003), (006) and (009) in the ZnAl-NAA-LDH diffractogram evidences the formation of the layered compound with the intercalation of NAA between the inorganic layers. The height and width of the peaks show a good structural organization of the synthesized LDH with no impurities. The basal spacing value calculated by means of the average basal peaks (00*l*) was 19.20 Å. This value is comparable to *d*-spacing values reported for ZnAl-NAA (20.5 Å) [15] and MgAl-NAA (19.5 Å) [26]. The ZnAl-NAA-LDH sample shows the parameter *a* = 3.06 Å, which is in agreement with a Zn/Al molar ratio of 2.

**Figure 2:**
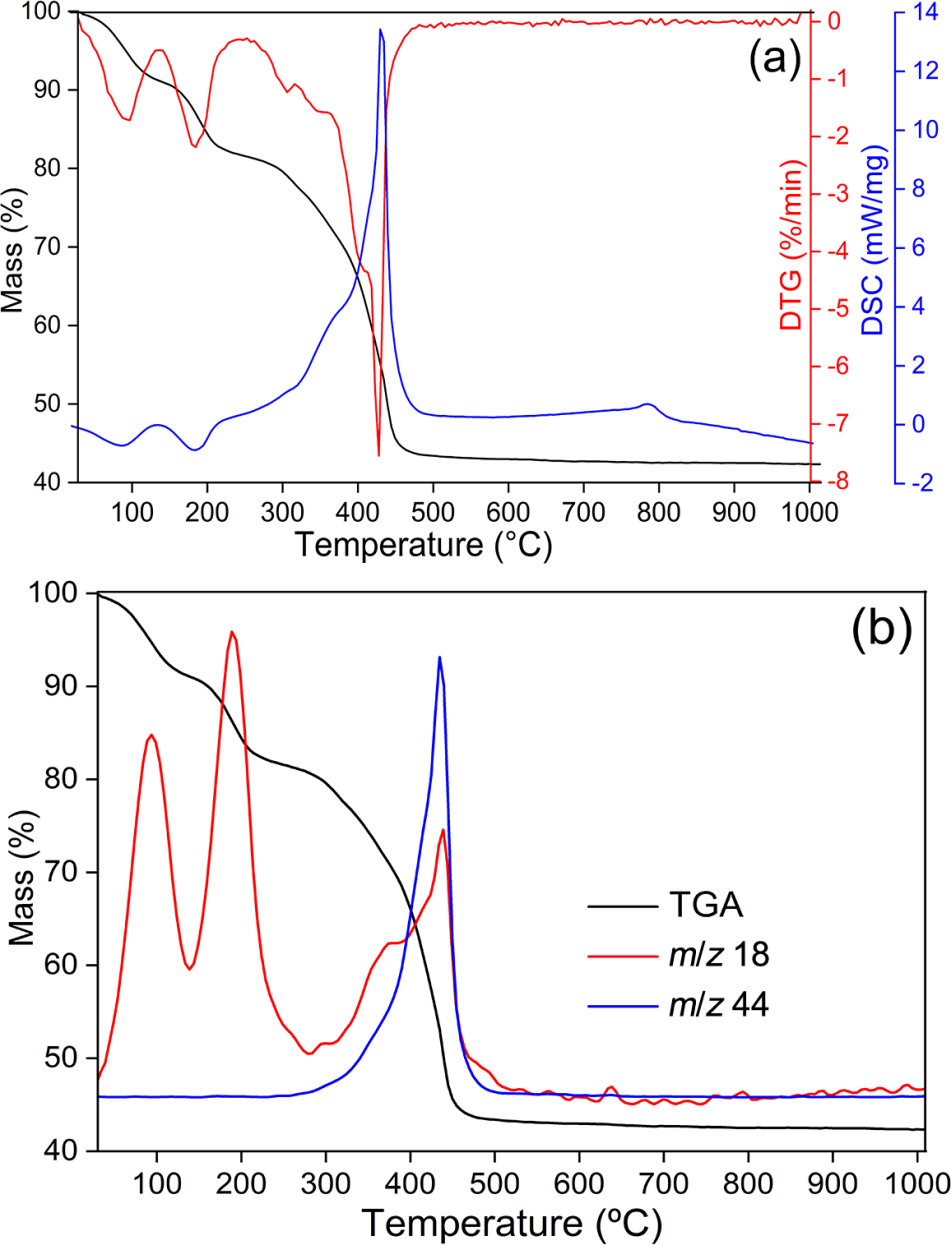
(a) TG-DSC curves and (b) TG-MS curves of ZnAl-NAA-LDH.

Figure 2 shows the thermogravimetric analysis-differential scanning calorimetry (TG-DSC) and thermogravimetric analysis mass spectrometry (TG-MS) curves of ZnAl-NAA-LDH, which evidence three main steps of mass loss. The first step occurs from room temperature to 155 °C (mass loss of 9.6%) and the release of a fragment with *m*/*z* 18 suggests sample dehydration. The second step from 155 °C up to about 260 °C (mass loss of 9.1% and *m*/*z* 18) is assigned to the dehydroxylation of the zinc–aluminium LDH layers [27]. Between 260 and 500 °C, the sample loses about 38% of mass and the fragments with *m*/*z* 18 and *m*/*z* 44 (water and carbon dioxide, respectively) are attributed to dehydroxylation and the thermal decomposition of the NAA anion.

Considering the hydration water amount (9.6%), the NAA content in ZnAl-NAA-LDH determined by electronic absorption spectrophotometry in the UV–vis region (37.1%) and the Zn/Al molar ratio of 2, the following formula is proposed for the hybrid material: Zn_2_Al(OH)_6_](NAA)·3H_2_O (calculated: 37.1% of NAA; 10.8% of H_2_O).

**Figure 3:**
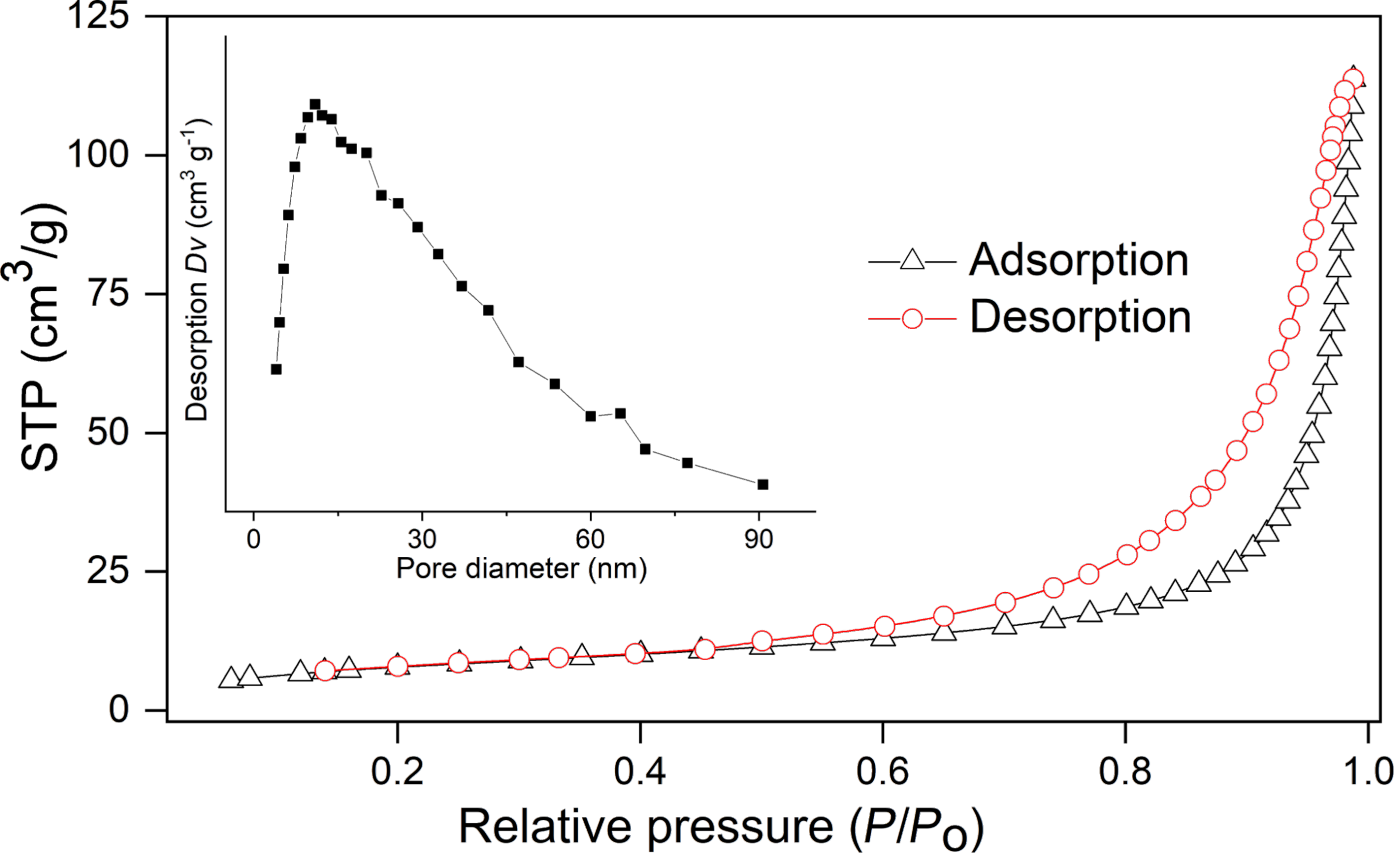
Nitrogen adsorption and desorption isotherms for ZnAl-NAA-LDH.

The morphology and porosity of LDHs can change depending on the synthesis method and also on the chemical compositions used to prepare these materials. These analyses are important to evaluate the release properties. The nitrogen adsorption and desorption isotherms for ZnAl-NAA-LDH presented in Figure 3, yielding values of specific surface area, volume, and average pore diameter of 30.0 mg^2^·g^−1^, 0.175 cm^3^·g^−1^, and 25.5 nm, respectively. The material shows a type-II isotherm, i.e., the graph is convex in relation to the relative pressure axis with no inflection point. Also, the material exhibits type-H1 hysteresis, a characteristic of porous materials with agglomerates, i.e., the particles are tightly aggregated with indefinitely coherent fragments [28–29]. The volume and average pore diameter values are characteristic of a mesoporous material.

**Figure 4:**
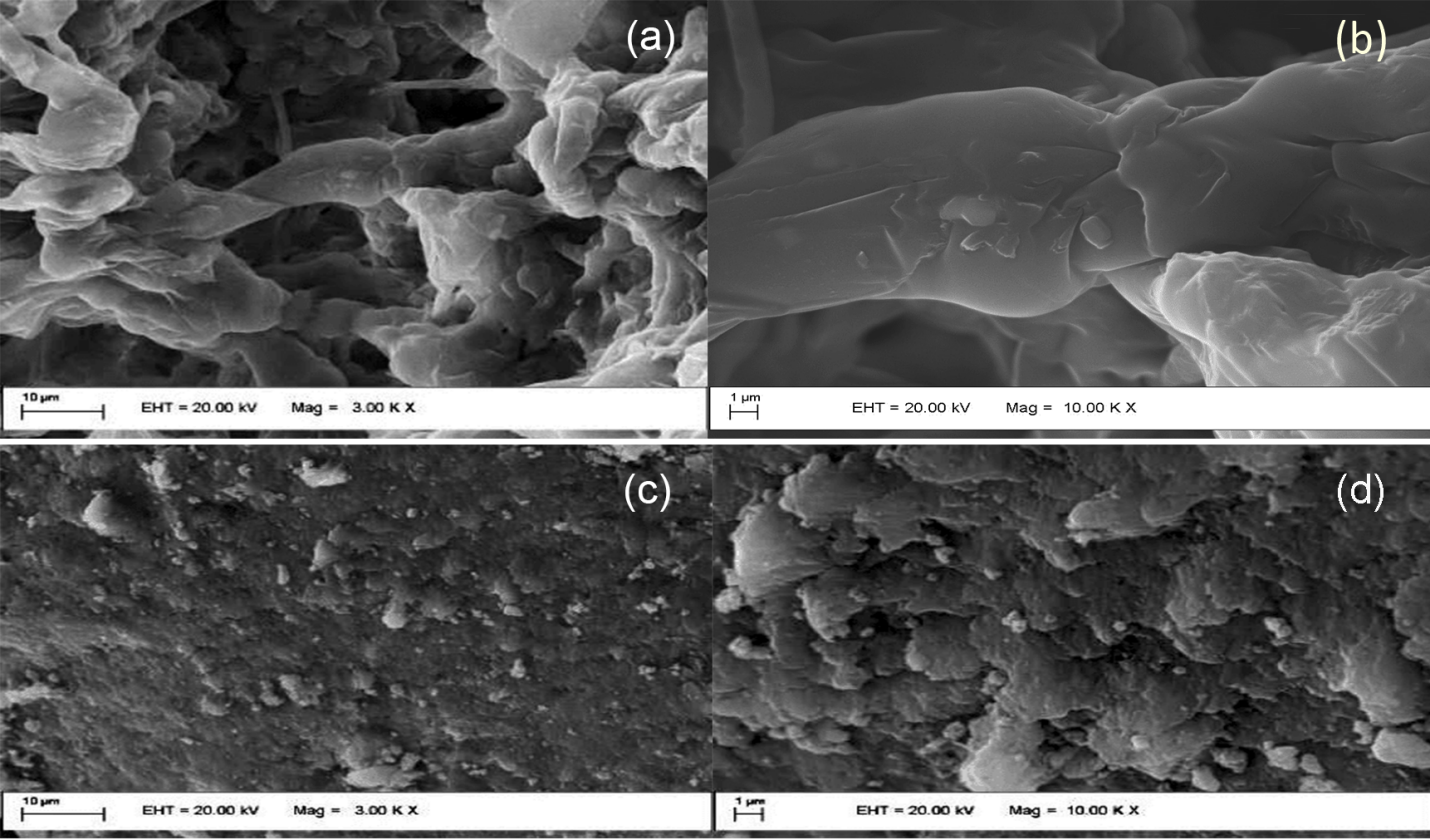
Representative SEM images of (a) NAA (3000× magnification), (b) NAA (10000× magnification), (c) ZnAl-NAA-LDH (3000× magnification), and (d) ZnAl-NAA-LDH (10000× magnification).

Scanning electron microscopy (SEM) images of NAA and ZnAl-NAA-LDH, presented in Figure 4, show the presence of particles with a smooth surface, probably resulting from the stacking of particles in the form of plates (Figure 4a,b). The ZnAl-NAA-LDH sample exhibits a surface in the form of compact plates (Figure 4c,d).

In vitro experiments to prove the efficiency of ZnAl-NAA-LDH as a slow-release matrix were performed in solutions with pH 4 and pH 7, and the release kinetics was also studied. See Supporting Information File 1 for complete experimental data.

### Germination rate and germination speed index

**Figure 5:**
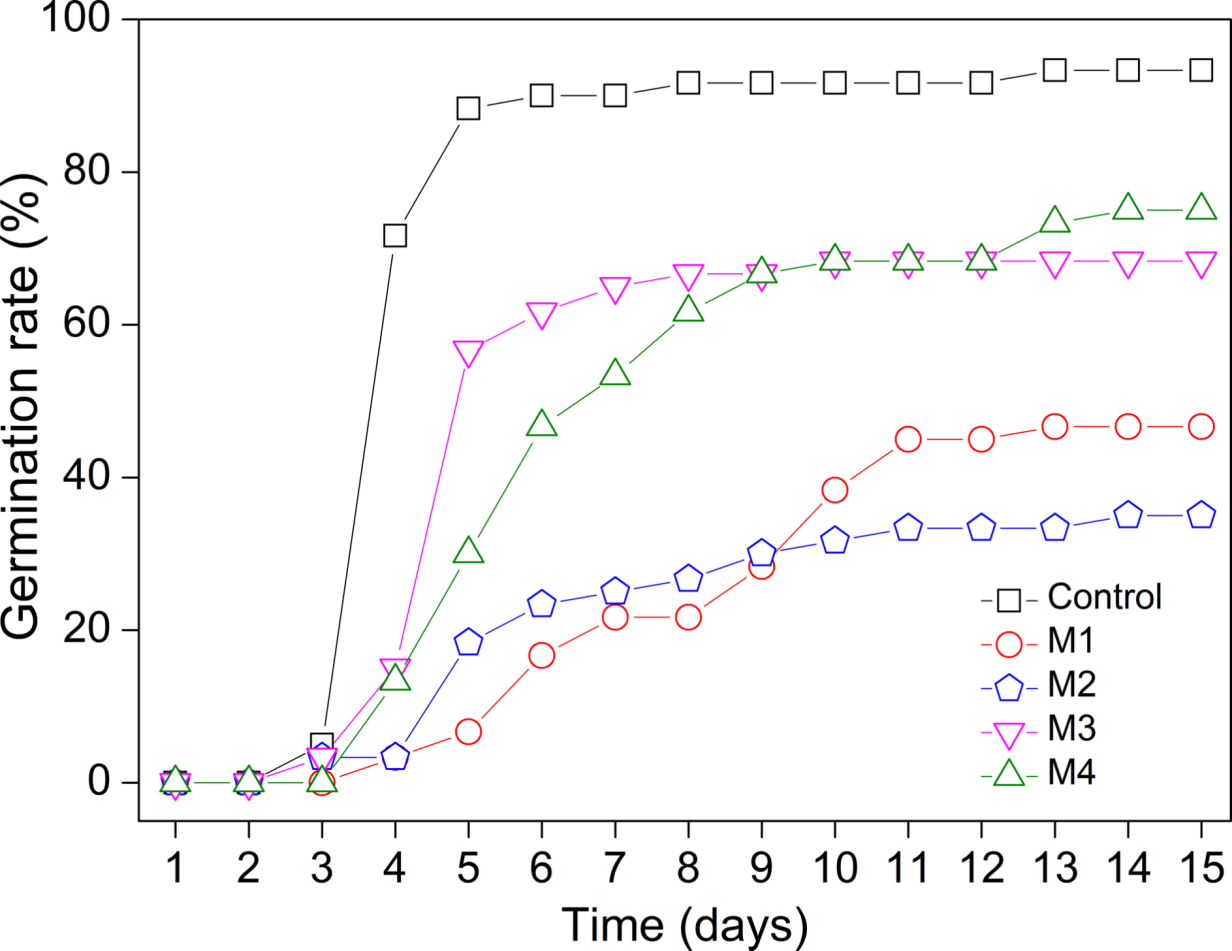
Germination rate in bean seeds covered with: control, M1 = neat alginate, M2 = alginate + ZnAl-CO_3_-LDH, M3 = alginate + NAA, and M4 = alginate + ZnAl-NAA-LDH.

The seed germination rate results are shown in Figure 5. After the final germination period of 15 days, the control experiment showed the highest percentage of germination (93.0%), followed by the seeds covered with the films M4 (alginate + ZnAl-NAA-LDH) (75.0%), M3 (alginate + NAA) (68.0%), M1 (neat alginate) (47.0%) and M2 (alginate + ZnAl-CO_3_-LDH) (35.0%). Both control and the M3 film reached a plateau of the percentage of germination after 6 days, until the end of the experiment after 15 days (Figure 5). For the M4 film, the maximum germination percentage was obtained 14 days after the bioassay assembly. For the treatments with the seeds covered with the films M1, M2 and M3, the maximum germination percentage was reached after 12, 11, and 9 days of the experiment, respectively.

In the seeds encapsulated with the M4 film, the maximum germination value and stability were reached after a longer period of time (14 days). This is related to the impeded water absorption by the seed due to the coating, which did not occur in the control. The coating of the seeds forms a barrier that decreases the absorption of H_2_O and the passage of O_2_. Consequently, the activation of enzymes, the respiration of the plant cells, and the cell duplication slow down. These factors delay the embryonic development of the plant and, subsequently, the quantity and speed of the germinated seeds are reduced [13,30]. Thus, the data shown in Figure 5 are satisfactory, as they prove a sustained release of the intercalated NAA, with the best results obtained for the seeds covered with the M4 film.

**Figure 6:**
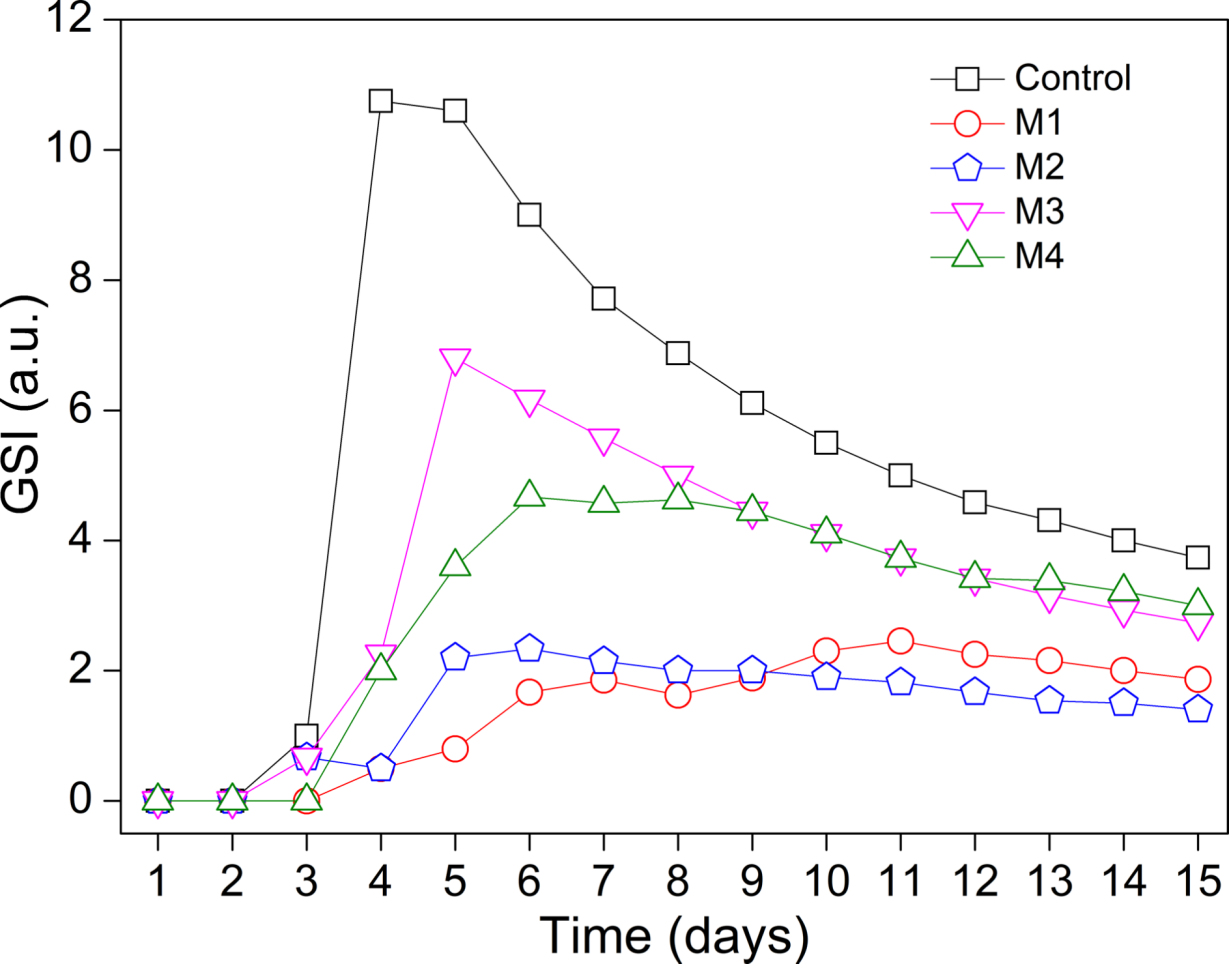
GSI in bean seeds covered with: Control, M1 = neat alginate, M2 = alginate + ZnAl-CO_3_-LDH, M3 = alginate + NAA and M4 = alginate + ZnAl-NAA-LDH.

The highest germination speed index (GSI) value obtained among all treatments was that for the control (Figure 6), evidencing a superior germinative behavior. The other treatments yielded GSI values in the following order: M3 > M4 > M2 > M1. The seeds covered with the M4 film had an intermediate GSI value (approximately 5) demonstrating that the film did not show phytotoxicity at the applied concentration (2.7 × 10^−3^ mg·L^−1^). Untreated seeds (control) and the seeds coated with the M3 film showed better performance than seeds coated with the M4 film. The coating acted as a physical barrier, hindering water absorption by the seeds, but also preventing the degradation of NAA, which is good for the seed, since the influence of NAA on the seed development was prolonged. This fact, evidenced by the germination percentage and the GSI test, confirmed the initial assumption that the seeds coated with the M4 film would present a lower GSI because of the modified release of auxin.

### Biometric analysis

**Figure 7:**
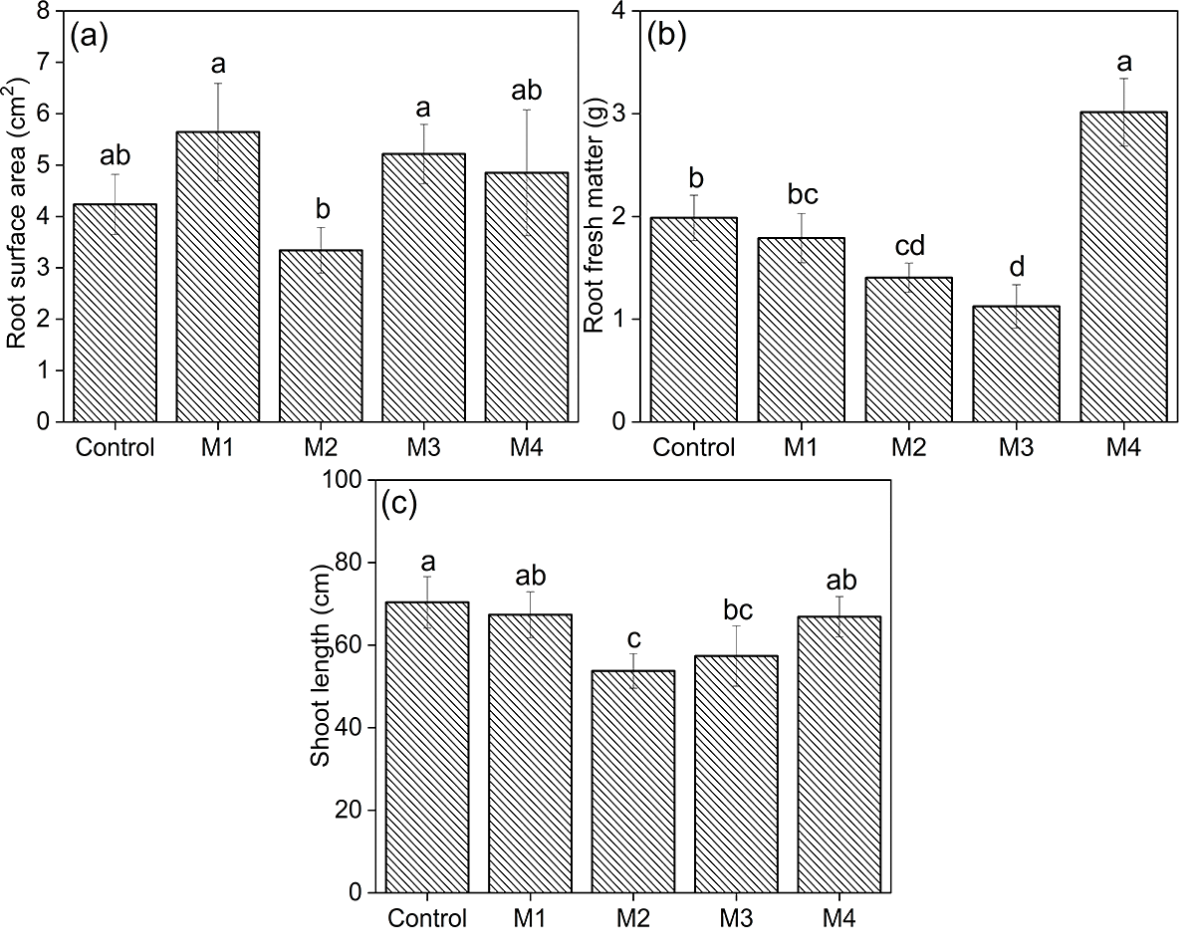
Values of: (a) root surface area, (b) root fresh matter and (c) shoot length for the beans being the seeds coated with: control, M1 = neat alginate, M2 = alginate + ZnAl-CO_3_-LDH, M3 = alginate + NAA e M4 = alginate + ZnAl-NAA-LDH. Averages followed by the same letter do not differ from each other by the Tukey test (*p* > 0.05).

According to the statistical results (Figure 7), the bioassay in pots with soil showed differences for the following analyzed criteria: (a) root surface area, (b) root fresh matter, and (c) shoot length. The results of root surface area (Figure 7a) showed the seed covered with the M3 and M1 films with the highest averages, differing only from the film M2. For the root fresh matter (Figure 7b) the treatment with the M4 film yielded the best result, differing from all others. Regarding the shoot length parameter (Figure 7c) the highest averages were expressed for the control, not differing from the seeds coated with the films M4 and M1.

To sum up, the seeds coated with the M4 film exhibited similar or superior results compared to the control, with enhanced growth and root formation. A close and precise relationship is observed between the results observed in vitro and in bioassays experiments. The treatment with M4 provides a slow and gradual release over time (Figure S1b, Supporting Information File 1). 25 days after planting, the M4 treatment precisely met the demand of the root regulator, culminating in an enhanced growth of root fresh matter (Figure 7b). The introduction of this new technology to sowing might lead to better a quality of seeds.

## Conclusion

The synthesis of a NAA-intercalated layered double hydroxide with a molar ratio Zn/Al = 2 by co-precipitation at constant pH value yielded a material with good crystallinity and phase purity. The basal spacing value observed for the material (19.5 Å) is characteristic of the intercalation of NAA between LDH layers. The auxin intercalated into ZnAl-NAA-LDH was thermally decomposed at temperatures above 270 °C. The hybrid material showed a low specific surface area (29.7 m^2^·g^−1^) and a mesoporous volume (0.17 cm^3^·g^−1^).

The seeds coated with the M4 film yielded a higher amount of bean root fresh matter than the M3 film and then control, confirming the postulate that the LDH-modified release prevented phytoxication through the PGR. The germination rate and GSI tests showed satisfactory results with the highest values close to that observed for the control, suggesting that the M4 film allowed NAA to stay longer in contact with the seed, while not being degraded, and to improve plant cell development.

In general, the use of polymeric films to cover seeds proved to be effective by promoting a better absorption of NAA, improving the physiological characteristics, and promoting a more prominent development of some organs of the plant. The introduction of this new technology to the sowing of bean seeds might lead to better a quality of the seeds, and consequently to a better development of the plant.

## Experimental

### Reagents

All reagents used in this work were of analytical purity: Zn(NO_3_)_2_·6H_2_O (96.0%) and Al(NO_3_)_3_·9H_2_O (98.5%) were provided by Dinâmica; 1-naphthaleneacetic acid (C_12_H_10_O_2_, 97.0%), acetonitrile (C_2_H_3_N, 99.8%), sodium alginate (NaC_6_H_7_O_6,_ >99.0%) and calcium nitrate (Ca(NO_3_)_2_, (99.0%) were supplied by Vetec; nitric acid (HNO_3_, 65.0%) was provided by F. Maia; sodium hydroxide (NaOH, 99.0%) was provided by Isofar and sodium hypochlorite (NaClO, 2.5%) was supplied by Merck. Water used for synthesis and washing of LDH was deionized by a Milli-Q^®^ system.

### Synthesis of the layered double hydroxide

The ZnAl-NAA-LDH hybrid was synthesized using a co-precipitation method at constant pH value. For the synthesis, 0.03 L of a solution containing 3.84 × 10^−3^ mol of Zn(NO_3_)_2_∙6H_2_O and 1.92 × 10^−3^ mol of Al(NO_3_)_3_∙9H_2_O were slowly added to 0.15 L of a solution containing 9.60 × 10^−3^ mol of NAA. During the synthesis, the suspension was maintained at pH 9.5 ± 0.5 by the slow addition of a 1.0 mol·L^−1^ solution of NaOH and the system was kept under constant agitation in a N_2_ atmosphere. The solid resulting from the synthesis was washed with H_2_O and dried under reduced pressure in a desiccator containing silica gel. The amount of NAA in the LDH was determined by electronic absorption spectrophotometry in the UV–vis region using the maximum absorption (λ_max_) at 280 nm [15].

The ZnAl-CO_3_-LDH material was synthesized for comparison in the bioassays. The sample was prepared by co-precipitation at constant pH value, according to a procedure adapted from Reichle [22]. For the synthesis, 0.25 L of a solution containing 3.54 × 10^−2^ mol of Zn(NO_3_)_2_∙6H_2_O and 1.77 × 10^−2^ mol of Al(NO_3_)_3_∙9H_2_O was prepared. Also, 0.25 L of a solution containing 7.03 × 10^−2^ mol of Na_2_CO_3_ was prepared. The carbonate solution was added under stirring to 1.0 L of water until pH 10. Then the solution containing the cations was slowly added to the solution containing the carbonate. The reaction mixture was maintained at pH 10 ± 0.5 by addition of a solution of 1.0 mol·L^−1^ NaOH. During the synthesis, the system was kept under constant agitation. After completion of the synthesis, the whole reaction medium was kept at rest for 12 h. The solid resulting from the synthesis was centrifuged and dried under reduced pressure in a desiccator containing silica gel.

### Coating of bean seeds with a polymeric film

Polymeric gels were prepared by dissolving sodium alginate in water for 2 h. The bean seeds were immersed in the gel and subsequently in a 5.0% solution of Ca(NO_3_)_2_ for 10 s to initiate the formation of films on the seeds surface. To homogenize the films, before being used in the bioassays, the seeds were dried in a drying oven at 25 °C for 2 h. The quantities of materials used for the preparation of the films named M1, M2, M3, and M4 are shown in Table 1.

**Table 1:** Composition of polymeric alginate films.

film	*V*_H2O_ (L)	alginate (mg)	ZnAl-CO_3_-LDH (mg·L^−1^)	NAA (mg·L^−1^)	ZnAl-NAA-LDH (mg·L^−1^)	weight %	
						NAA	LDH

control	—	—	—	—	—	—	—
M1	0.025	500	—	—	—	—	—
M2	0.025	500	1.0 × 10^−3^	—	—	—	0.04
M3	0.025	500	—	1.0 × 10^−3^	—	0.04	—
M4	0.025	500	—	—	2.7 × 10^−3^	0.04	0.11

### Bioassays

**Figure 8:**
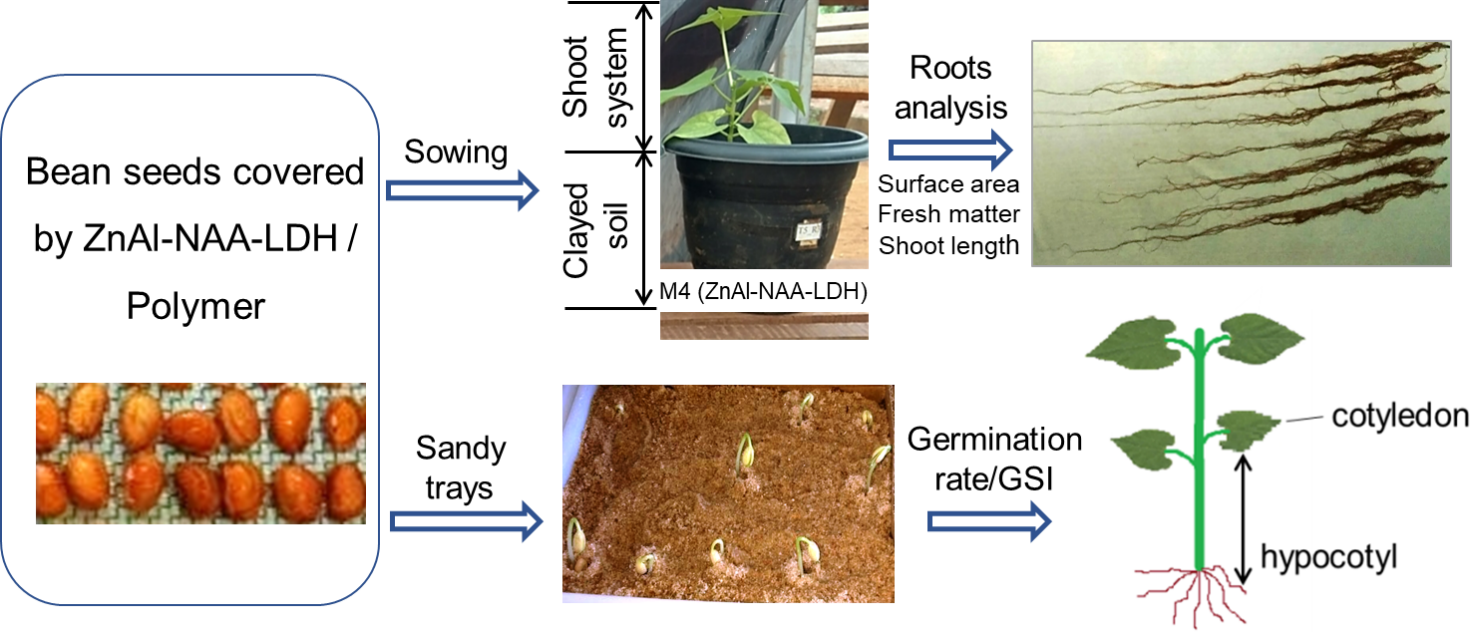
Scheme of the bioassays.

The bioassays were carried out in a greenhouse with plants cultivated in pots of 3.0 dm^−3^ filled with clayey soil collected at the geographic coordinates 19°12'34.3"S and 46°07'57.1"W, which present the chemical composition shown in Table 2. A scheme of the bioassays is presented in Figure 8.

**Table 2:** pH values, macronutrients, micronutrients, organic matter and organic carbon contents of the soil used in the bioassays.

soil	

pH in water	6.0
pH in CaCl_2_ solution	5.1

macronutrients	(cmol_c_·dm^−3^)

K	0.03
Ca	2.2
Mg	0.4
Al	0.0

micronutrients	(g·dm^−3^)

B	2.6 × 10^−4^
Cu	7.0 × 10^−4^
Fe	2.2 × 10^−2^
Mn	8.0 × 10^−4^
Zn	7.0 × 10^−4^
O. M. (organic matter)	32.0
O. C. (organic carbon)	18.6

In the bioassays were evaluated: (i) germination rate and seed germination speed index; and (ii) a plant biometric analysis including root area, root fresh matter and shoot length of the plants.

### Germination rate and germination speed index

The germination rate [30] and GSI [31] tests were conducted in a laboratory at 25 °C. The bean seeds presenting 13% of water content on average were disinfested using a 2.5% sodium hypochlorite solution for 2 min and then washed with deionized water. After coating the seeds with the polymeric films, as previously described, sowing was performed in sand trays as recommended by the Brazilian Rules for Seed Analysis [30]. Fifteen seeds were used for each trial and before sowing they were treated as described in Table 1 (except the control). Then they were planted in sand trays (Figure 8). The assays were performed in quadruplicate totalizing 20 trays. The number of germinated seeds was evaluated daily for a period of 15 days. The germination criterion was the emergence of the cotyledon, that is, the first leaves that emerge from the embryo erupting during the germination of the seeds, with the consequent emergence of hypocotyl, which is the part of the axis of the embryo or seedling located between the insertion point of the cotyledon and the one in which the radicle begins [32].

The calculation of the GSI was performed using an adaptation of the formula described in [31]:

[1] $$\text{GSI}=\frac{G1}{N1}+\frac{G2}{N2}+...+\frac{Gn}{Nn},$$

where *G*1, *G*2 and *Gn* are the number of normal seedlings obtained in the first, second and last counts, respectively, and *N*1, *N*2, and *Nn* are number of days after sowing at the first, second, and last count, respectively.

### Biometric analyses

Biometric analyses were performed for five samples. One was the control without polymer coating. The other four samples were coated with pure alginate; alginate and ZnAl-CO_3_-LDH, alginate and NAA, or alginate and ZnAl-NAA-LDH, with four replications. Six bean seeds previously coated with polymer (as described in Table 1) were sown in each pot at a depth of 3.0 cm. 10 days after sowing, four plants were thinned, maintaining only two plants per pot for later analyses performed 25 days after planting. Root area analyses were performed with the software SAFIRA – *Software de análise de fibras e raízes por imagem* (fibre and root analysis software by image) [33]. Root fresh matter determination was performed with the aid of a high-precision analytical scale (Shimadzu AUY 220). The length of the shoot of the plants was measured directly with a high-grade ruler (1:1.000).

### Characterizations techniques

Powder X-ray diffraction patterns (XRD) were recorded in a Shimadzu X-ray Diffractometer XRD-6000 mode using Cu Kα_1_ radiation (λ = 1.5406 Å), 40 kV, 40 mA, sweep range 2θ from 2° to 70° with a scan step of 0.02°/s. Electronic absorption spectra were acquired in an equipment Thermo Scientific model Evolution 300.

The morphology of the LDH samples was analyzed by SEM using a Carl Zeiss scanning microscope, model EVO 50. The sample was supported on the sample holder by dispersion of the powder on conductive double-sided adhesive tape. The samples were gold-coated before the measurements using a Sputter BAL-TEC, MED 0.50.

Mass-coupled thermal analyses (TG-DSC-MS) were performed on a Netzsch thermoanalyzer model TGA/DSC 409 PC – Luxx coupled to an Aëolos 403 C mass spectrometer, using alumina crucibles and a heating rate of 10 °C/min under synthetic air flow of 50 cm^3^/min from ambient temperature up to 1000 °C.

Surface area analysis was determined by measuring adsorption/desorption isotherms of N_2_ at −196 °C, using a surface area analyzer Micromeritics Model ASAP 2020n. The samples were heated at 80 °C for 48 h under reduced pressure before adsorption measurements. The specific surface area was determined by the BET method.

### Statistical analysis

The determinations of biometric analysis were performed a completely randomized experimental design, with analysis of variance tests, homoscedasticity and comparison of averages for each treatment. The data obtained were subjected to analysis of variance and the averages observed compared by the Tukey test with 5.0% significance.

## Supporting Information

File 1 In vitro release experiments.
