# Supplementary material for: Plant growth regulation by seed coating with films of alginate and auxin-intercalated layered double hydroxides
Source: Beilstein J Nanotechnol. 2020 Jul 24;11:1082–91. doi: 10.3762/bjnano.11.93 (PMC7385381; doi:10.3762/bjnano.11.93)
Supplement: File 1 — In vitro release experiments. [file Beilstein_J_Nanotechnol-11-1082-s001.pdf]

BEILSTEIN JOURNAL OF NANOTECHNOLOGY

Supporting Information

for

Plant growth regulation by seed coating with films of alginate and auxin-intercalated layered double hydroxides

Vander A. de Castro, Valber G. O. Duarte, Danúbia A. C. Nobre, Geraldo H. Silva, Vera R. L. Constantino, Frederico G. Pinto, Willian R. Macedo and Jairo Tronto

Beilstein J. Nanotechnol. **2020,** *11,* 1082–1091. doi:10.3762/bjnano.11.93

In vitro release experiments

License and Terms: This is a supporting information file under the terms of the Creative Commons Attribution License (http://creativecommons.org/licenses/by/4.0). Please note that the reuse, redistribution and reproduction in particular requires that the authors and source are credited.
The license is subject to the *Beilstein Journal of Nanotechnology* terms and conditions: (https://www.beilstein-journals.org/bjnano)

Controlled release of NAA into aqueous solutions

Experimental

The in vitro release experiments with neat NAA and ZnAl-NAA-LDH were performed in solutions with pH 4 and pH 7. The pH value of the solutions was kept constant during the experiments by the addition of HNO_3 (0.15 mol·L^{-1}) and NaOH (0.10 mol·L^{-1}). The suspensions of neat NAA and ZnAl-NAA-LDH were prepared by dissolving 1.0×10^{-2} mg of neat NAA and 2.7×10^{-3} mg of LDH, respectively, both in 0.1 L H_2O. The amounts of NAA used in these experiments were equated.

The samples were kept under constant agitation at 80 rpm and 25 °C over 24 h. The experiments were performed in triplicate. During the release process, aliquots of 5.0×10^{-3} L of the samples were taken at predetermined time intervals and filtered through quantitative filter paper. Then, the pH values were measured and if necessary adjusted as described above. The amount withdrawn in the aliquots was replenished keeping the volume constant. The amount of NAA released into the suspended samples was determined by UV–vis spectrophotometry [1].

Results

The in vitro kinetic release profiles shown in Figure S1 do not fit the Higuchi model [2] while the profiles shown in Figure S2 fit the Higuchi model [2]. Neat NAA and ZnAl-NAA-LDH showed faster accumulated release rates in the first hour for both cases, fitted to the model ($t^{0.5}$ = 1.0 h) and not fitted to the model (t = 1.0 h), exhibiting a difference in the profile of the curve but yielding the same release range. The equilibrium for neat NAA was reached after 3 h (not fitted to the model) and ($t^{0.5}$ = 1.7 h) (fitted to the model) for both pH values. ZnAl-NAA-LDH yielded a slow and gradual

release of NAA. The kinetic study showed that ZnAl-NAA-LDH exhibited lower release values than neat NAA. In both suspension samples, pH 4 and pH 7, about 30% of the intercalated NAA was released after t = 24 h (not fitted to the model) and $t^{0.5}$ = 5.0 h (fitted to the model), while 60% of pure NAA was released in the same period for both cases studied.

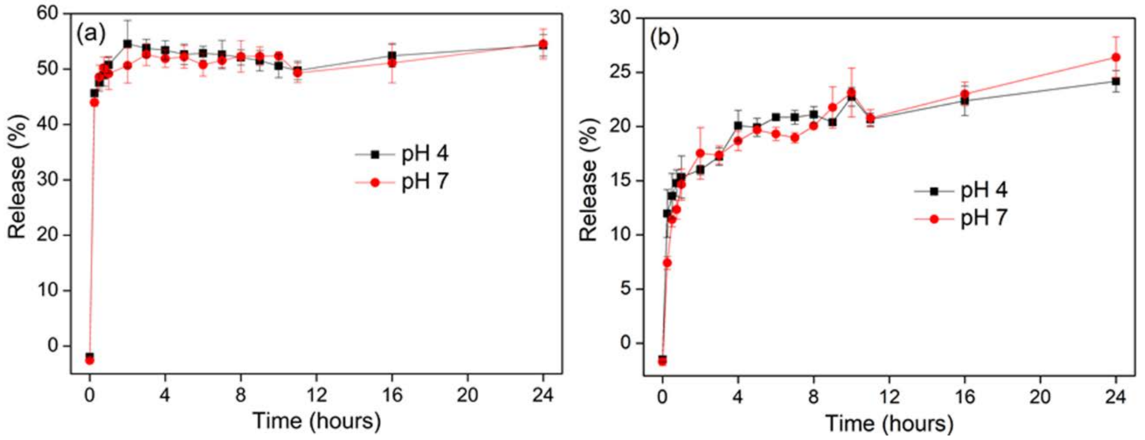

Figure S1: Release profiles not fitting the Higuchi model at pH 4 and pH 7 for (a) neat NAA and (b) ZnAl-NAA-LDH.

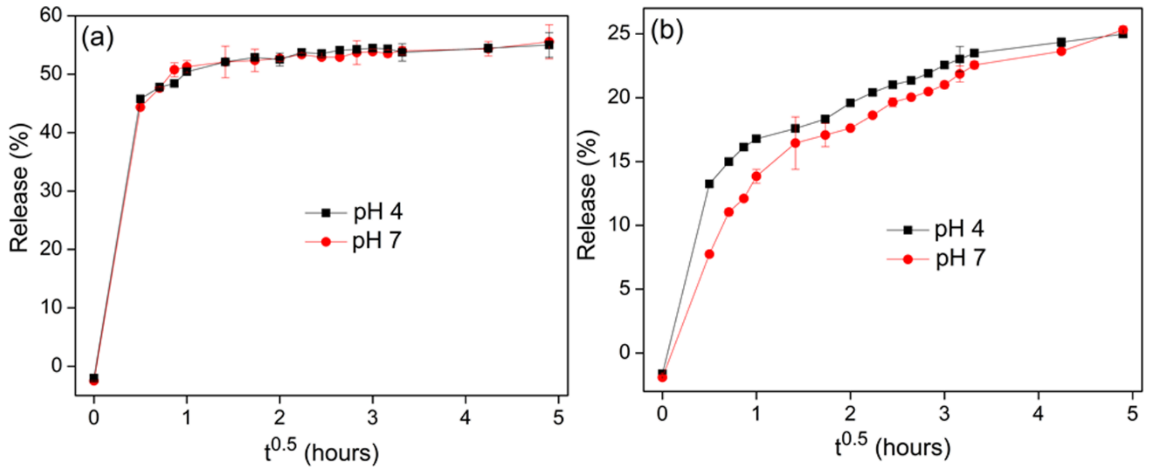

Figure S2: Release profiles fitting the Higuchi model at pH 4 and pH 7 for (a) neat NAA and (b) ZnAl-NAA-LDH.

The results obtained here are similar to those obtained by Hussein et al. [1] using LDH as a host matrix in aqueous solutions with different pH values. The authors described that in aqueous solutions with low initial pH (adjusted by the addition of HNO_3), the high concentration of NO_3^- ions resulted in the exchange of NAA ions by NO_3^- ions into the inorganic layers of the LDH. As a result, NO_3^- ions were incorporated into the ZnAl-NAA-LDH interlayer space and, at the same time, the deprotonation of NAA in the solution occurred to a smaller extent. When starting the experiment, the ion exchange process is fast. When a larger species such as NAA is exchanged for a smaller anion, such as NO_3^-, a decrease in basal spacing occurs and this phase transformation hinders the further release of NAA. As the reaction goes on, smaller and larger basal spacings coexist in the same crystal. As a result of this new phase, a barrier forms between ZnAl-NAA-LDH and the aqueous solution and, consequently, the released NAA rate for the aqueous solution is lower [1]. This is why the amount of released NAA from ZnAl-NAA-LDH at pH 4 is greater than the released amount at pH 7. Figure S2b shows a material profile with the typical behavior described above. The results were presented with data from the whole contact period of neat NAA and ZnAl-NAA-LDH with the aqueous solutions, where $t^{0.5} = 5.0$ is equivalent to a total time of 24 h.

References

[1] Bin Hussein, M. Z.; Zainal, Z.; Yahaya, A. H.; Foo, D. W. V. *J. Controlled Release* **2002,** *82,* 417–427. doi:10.1016/S0168-3659(02)00172-4

[2] Costa, P.; Lobo, J. M. S. *Eur. J. Pharm. Sci.* **2001**, *13*, 123–133. doi:10.1016/S0928-0987(01)00095-1
